# Endoscopic endonasal transpetroclival approach for recurrent bilateral petroclival meningioma

**DOI:** 10.3171/2022.1.FOCVID21229

**Published:** 2022-04-01

**Authors:** Yuki Shinya, Masahiro Shin, Hirotaka Hasegawa, Satoshi Koizumi, Taichi Kin, Kenji Kondo, Nobuhito Saito

**Affiliations:** 1 Department of Neurosurgery, The University of Tokyo Hospital, Tokyo, Japan;; 2 Department of Neurosurgery, Teikyo University Hospital, Tokyo, Japan; and; 3 Department of Otorhinolaryngology, The University of Tokyo Hospital, Tokyo, Japan

**Keywords:** endoscopic skull base surgery, skull base tumor, meningioma

## Abstract

The authors performed an endoscopic endonasal transpetroclival approach for recurrent bilateral petroclival meningioma, with the aim of sufficient tumor resection with cranial nerve functional preservation. The tumor was sufficiently removed with excellent postoperative course. Petroclival meningioma, especially located in the medial region with dural attachment of the clivus, is considered a good indication for this approach. Recurrent tumors after radiotherapy often have strong adhesion to the brainstem and basilar artery; therefore, careful assessment of whether or not tumor detachment is possible is essential. The endoscopic endonasal transpetroclival approach is an acceptable, less-invasive treatment for petroclival tumors.

The video can be found here: https://stream.cadmore.media/r10.3171/2022.1.FOCVID21229

## Transcript

This is a case of recurrent bilateral petroclival meningioma treated with an endoscopic endonasal transpetroclival approach.

### 0:30 Patient Presentation.

A 53-year-old woman presented with recurrent bilateral petroclival meningioma, resulting in left facial sensory disturbance, abducens nerve palsy, facial palsy, hearing loss, and truncal ataxia. She had transcranial resection via a combined petrosal approach followed by radiosurgery for the primary tumor 25 years ago. On magnetic resonance imaging (MRI), the tumor extended widely from the dorsum sellae to the lower clivus, internal auditory canal, and jugular foramen, resulting to the strong compression of the brainstem. We planned an endoscopic endonasal transpetroclival approach that aimed for a safe maximal resection with functional preservation of the cranial nerve.

### 1:23 Surgical Setup.

Feeder embolization of the meningohypophyseal trunk, middle meningeal artery, and ascending pharyngeal artery was performed before surgery. Preoperative vertebral angiography showed that there was no irregular shape on the bilateral vertebral arteries and basilar artery, and arterial involvement was not suspected. During the endoscopic transpetroclival approach, we can directly visualize if the tumor is adherent to the basilar artery or vertebral arteries. In case that tumor invaded into those major arteries, we have to give up total resection and balance the safety and the level of resection. After induction of general anesthesia, lumbar drainage was placed, and the patient was placed in the supine position and fixed with a Mayfield 3-point head holder, with the head slightly up and rotated minimally to the right and tilted to the left. The 4-mm straight and 30° endoscopes were mainly used during the resection, and a 70° lens was used to look in the tumor margins. Intraoperative monitoring of the third nerve through lower cranial nerves, motor evoked potential (MEP), somatosensory evoked potential (SEP), and auditory brainstem response (ABR) were supportively used. Stealth navigation system and robotic endoscope holder were also used.

### 2:55 Transpetroclival Approach.

The tumor is approached through the bilateral nostrils. First, we enter the sphenoid sinus and expose the sella turcica, clival recess, and bilateral internal carotid prominence. Next, the clival bone and the sphenoid floor are drilled out. Bleeding from the basilar plexus is controlled using a thrombin-gelatin matrix. The clival dura mater at the tumor attachment is cauterized entirely using a bipolar electrocautery; this leads to devascularization prior to the intradural procedures.

### 3:41 Tumor Resection.

The clival dura is incised using a microsurgical blade and microscissors; the dura in this area is very thick. Since the tumor is exposed just below the dura, the dura is dissected off of the tumor to expose the ventral surface of the tumor. After debulking the tumor, the left abducens nerve is seen here on the left side of the patient. The tumor is gently dissected off of this nerve and removed. After resection of the central part of the tumor, our attention is shifted to the right side of the surgical field (which would be the left side of the patient), where the tumor is cauterized and dissected from the left petrous bone. The lower cranial nerves are then exposed posteriorly. The tumor is tightly adherent to the basilar artery and the anterior surface of the brainstem; therefore, we carefully dissect the tumor in this area. On the left side of the surgical field (which would be the right side of the patient), the tumor is also cauterized from the medial side of the petrous dura while moving to the deeper side. Tumor removal continued, and the bilateral vertebral arteries and the anterior aspect of the medulla are exposed. The right facial and vestibular nerves are also exposed in this area. A significant portion of the tumor is removed. Thereafter, the lateral edge of the tumor gradually comes into the surgical field, and the left abducens nerve is now exposed. The tumor is dissected off of the nerve, and the tumor of this part is removed. Sufficient tumor resection is achieved at the end of the procedures (Simpson grade IV).

### 6:02 Closure.

Gelfoam is placed in the surgical cavity to make up for the arachnoid membrane. The inlay fascia lata is placed so it completely covers the dural defect from the inner side, and an onlay fascia is placed to cover up the dural defect from the outer side, which is sealed with fibrin glue. Pieces of fat are then attached to the margins of the dural defect and sealed with fibrin glue. A sinus balloon is inflated to fix the tissues we placed. The left and right nasal septal mucosa are restored, and the surgery is completed. For closure, the multilayered fascial reconstruction is performed and a pedicled nasoseptal flap is not used. In this approach, the tumor invaded in bilateral regions are treated in a single surgical procedure.

### 7:00 Postoperative Course.

The patient’s postoperative course was uneventful with significant improvement of truncal ataxia. Regarding cranial nerve function was as follows. The left facial sensory disturbance and hearing loss was unchanged. The left abducens nerve palsy was transiently and mildly worsened after the surgery but completely resolved within 6 months. The left facial palsy also improved. Adequate tumor removal was confirmed on the postoperative MRI. The histological examination showed meningothelial meningioma with a Ki-67 index of 2%. Upon follow-up at 1 year postsurgery, the patient has no sign of tumor recurrence.

### 7:53 Learning Points.

The endoscopic transnasal transpetroclival approach is an adequate operative intervention for the tumor penetrating the clivus to the prepontine cistern that severely compressed the brainstem and extended laterally to the petroclival region.^1–9^ However, application of this approach should be discussed considering the size of the approach window (clival recess) in contrast with the size, lateral extension of the tumor, and expected tumor consistency.^5^ It is important to carefully dissect the tumor from the pia mater of the brainstem piece by piece. Recurrent tumors after radiotherapy often have strong adhesion to the brainstem and basilar artery; therefore, it is important to carefully assess if tumor detachment is possible in the procedure.

## Disclosures

The authors report no conflict of interest concerning the materials or methods used in this study or the findings specified in this publication.

## Author Contributions

Primary surgeon: Shin. Assistant surgeon: Shinya, Hasegawa, Kondo. Editing and drafting the video and abstract: Shin, Shinya, Kin, Kondo. Critically revising the work: Shin, Hasegawa, Koizumi. Reviewed submitted version of the work: Shin, Shinya, Hasegawa, Koizumi, Kondo, Saito. Approved the final version of the work on behalf of all authors: Shin. Supervision: Shin, Saito.
